# Self-care methods use for acne treatment among health science students

**DOI:** 10.1016/j.rcsop.2025.100601

**Published:** 2025-04-03

**Authors:** Sari Taha, Manal Taha, Sa’ed H. Zyoud

**Affiliations:** a An-Najah Global Health Institute, An-Najah National University, Nablus 44839, Palestine; b Department of Public Health, An-Najah National University, Nablus 44839, Palestine; c Médecins Sans Frontières France, 15 Street, Nablus, Palestine; d Department of Clinical and Community Pharmacy, Faculty of Pharmacy, An-Najah National University, Nablus 44839, Palestine; e Poison Control and Drug Information Center (PCDIC), An-Najah National University, Nablus 44839, Palestine; f Clinical Research Centre, An-Najah National University Hospital, Nablus 44839, Palestine

**Keywords:** Complementary and alternative medicine, Acne, Acne severity, Dermatology, Health-seeking behavior, Over-the-counter medications

## Abstract

**Introduction:**

The use of self-care methods, such as over-the-counter (OTC) products and complementary and alternative medicine (CAM), is common along the acne care pathway. This study aimed to explore self-care methods for acne and assess their associations with acne severity.

**Methods:**

This cross-sectional study was conducted among university students in health programs. Convenience sampling was used to invite participants to complete a survey containing sociodemographic, clinical, and self-care-related questions. Acne diagnosis and severity were evaluated by a physician via the Global Acne Grading System (GAGS). A multivariate regression model was used to analyze the associations between variables.

**Results:**

The final sample comprised 190 participants, with 24.2 % males and 70.8 % females. Most participants had mild acne (77.4 %) and reported positive family histories (82.1 %). Approximately one-third used OTC products (31.1 %), and nearly two-thirds used CAM (62.6 %). The most frequently used OTC products were facial cleansers (48.2 %), followed by creams and moisturizers (30.7 %) and cleansing soap (21.1 %). A lack of acne severity was the primary reason for the use of CAM. Social media (46.7 %) and the internet (46.2 %) were the most frequently reported sources of information. Acne severity was associated with OTC product use (*p* *=* 0.009) and the duration of acne (*p* < 0.001). Furthermore, OTC product use was associated with receiving a professional diagnosis (*p* < 0.001).

**Conclusions:**

This study identified the most common OTC products and CAM used in acne and demonstrated an association between OTC product use and acne severity. Future studies should explore discussions on self-care methods in clinical consultations and the timing of using these methods throughout the care pathway. Integrating shared decision-making in clinical practice and tailoring educational interventions to patient preferences and communication channels may encourage the safe and effective use of self-care methods.

## Highlights

• Using complementary and alternative medicines is common among acne patients.
• Acne patients resort to online sources for information on nonprescription products.
• Using nonprescription products and acne duration are linked to severity.
• Using self-care methods should be discussed in clinical consultations.
• Management practices of young acne patients can be addressed using online platforms.

## List of abbreviations

**Table:** 

BMI	body mass index
CAM	complementary and alternative medicine
OTC	over the counter
NNU	An-Najah National University (NNU)
GAGS	Global Acne Grading System
WHO	World Health Organization

## 1 Introduction

Acne vulgaris is a chronic disease characterized by noninflammatory comedones and inflammatory lesions, including papules, pustules, cysts and nodules. It can lead to postinflammatory pigmentation and scarring, including depressed, keloid, and hypertrophic scars.^1^ In addition, acne is associated with reduced quality of life, unemployment, depression, and anxiety.2., 3, 4 Management strategies include the use of pharmaceutical therapies such as oral antibiotics; lifestyle modifications; and alternative options, such as lasers, blue light, photodynamic therapy, and chemical exfoliation.^5^, ^6^, ^7^, 8., 9.

Patient perceptions shape self-care practices in acne management and influence treatment choices along the acne care pathway.^10^ Some patients perceive acne as self-limiting and attempt lifestyle modifications, such as frequent washing and increased water intake, which may delay or prevent professional consultation.^11^ Additionally, acne is often considered a cosmetic problem,^12^ prompting the use of over-the-counter (OTC) products, which are obtainable without a prescription.^13^^,^^14^ Decker and Garber classified OTC acne products into cleansers, leave-on products, mechanical treatments, essential oils, and vitamins.^15^ In addition to OTC products, patients may use complementary and alternative medicine (CAM), which includes therapeutic approaches outside standard medical care, such as herbs, home remedies, supplements, and traditional medical systems.^16^ Notably, acne patients use CAM more frequently than those with other dermatological conditions.^17^^,^^18^ Green tea, *aloe vera*, and certain dietary modifications are among the CAM approaches that have demonstrated efficacy in acne, albeit with inconclusive evidence.19., 20, 21, 22

Personalized care, which integrates patient autonomy and preferences into treatment decisions,^23^^,^^24^ is key for acne management. This is because the disease has diverse presentations and psychosocial impacts, necessitating a tailored approach for the individual patient.^25^^,^^26^ Moreover, the chronicity of acne, its slow response to treatment, and the potential for relapses require ongoing care and adherence to treatment.^27^^,^^28^ In chronic skin diseases, treatment adherence is influenced by multiple factors, including treatment expectations, cost, lifestyle impact, disease chronicity, severity, visibility, and physician–patient communication.^29^

Conventional acne management relies on a physician's clinical assessment and treatment selection.^30^^,^^31^ When the patient is placed as a passive recipient of care, this traditional approach may overlook the factors influencing adherence to treatment, potentially leading to treatment failure.^25^ Alternatively, the Personalizing Acne: Consensus of Experts (PACE) provides management recommendations that emphasize personalized care in acne.^25^^,^^32^ In this alternative approach, discussion and selection of treatment options are informed by the patient's values, concerns, and preferences.^25^^,^^32^ Importantly, self-care fits this personalized approach, improving patient adherence and treatment outcomes, especially given the chronic, relapsing course and psychosocial impact of acne.^27^^,^^32^

Globally, several studies have explored the health-seeking behaviors of acne patients and their relationship with acne severity.^33^, ^34^, 35., 36. In Palestine, previous studies conducted among university students found that self-care practices are common in acne. Patients use various forms of OTC products and CAM, including medications that are obtainable without a prescription in Palestine but typically require one elsewhere, such as oral antibiotics.^37^^,^^38^ In one of these studies, however, neither acne diagnosis nor its severity was assessed by a physician, relying on self-diagnosis and assessment.^37^ This does not distinguish past from present diagnoses, possibly introducing measurement bias and explaining the high prevalence rate.^37^ Additionally, this study largely focused on dietary factors and quality of life related to acne without exploring the acne care pathway.^37^ Another study was primarily descriptive in nature, focusing on exploring trends of CAM use for acne.^38^ Moreover, none of these studies assessed potential self-care associations with either acne severity or seeking professional care, and both included students with and without acne.^37^^,^^38^ Understanding variations in health-seeking behavior can highlight self-care trends and inform personalized management, patient education, and targeted interventions. This study aimed to explore self-care methods, including OTC and CAM use, among university students diagnosed with acne in Palestine and assess the associations between self-care methods and acne severity.

## 2 Methods

### 2.1 Study design and setting

An analytical, cross-sectional study was conducted at the Faculty of Medicine and Health Sciences, An-Najah National University (NNU), located in Nablus, Palestine, between April and August 2023.

### 2.2 Population and inclusion and exclusion criteria

Students who were diagnosed with acne and enrolled at the Faculty of Medicine and Health Sciences were eligible for participation. Patients with medication-induced acne (those who used anabolic steroids,^39^ oral contraceptive pills, corticosteroids,^40^ lithium,^41^ or phenytoin^42^) and those who had received a diagnosis of polycystic ovarian syndrome, hormonal imbalance, or pregnancy were excluded from the study. A nonprobability convenience sampling technique was used to approach students during lectures and invite them to participate in the study. The sample size was estimated to be 380 via the equation *n = Z.*^2^*P. (1-P)/d,*^2^ where *n* is the sample size, *Z* is the value determined by the level of confidence (at 95 %), *P* is the expected prevalence of acne according to previous studies, and *d* is the precision level (at 5 %).^43^ The prevalence was estimated to be 45 % on the basis of a study conducted in Jordan.^44^ Assuming a dropout rate of 10 %, the following equation yields an adjusted sample size of 422: *N* = *n*/1-*r*, where *N* is the adjusted sample size and *r* is the expected dropout rate.^45^

### 2.3 Data collection and variables

#### 2.3.1 Acne diagnosis and severity assessment

Acne was diagnosed by a general practitioner, ensuring the privacy and confidentiality of the participants. All invited participants were assessed for acne by the same clinician. Only participants who were diagnosed with acne were enrolled in the final study. Acne severity was assessed via the Global Acne Grading System (GAGS), which was developed by Doshi et al.^46^ To increase the validity of the study, the examiner was trained to use the GAGS tool to diagnose and assess acne severity.^46^ The score calculation depends on the type and location of acne lesions. Initially, the GAGS assigns a score for every involved location (2 for forehead, 2 for right cheek, 2 for left cheek, 1 for nose, 1 for chin, 3 for chest and upper back), and another score according to the most severe lesion in that location (1 for comedones, 2 for papules, 3 for pustules, and 4 for nodules/cysts). The local grade of an involved location is calculated by multiplying the location score by that assigned to the most severe lesion in the respective location. The global grade is calculated by summing the local grades for each location, which can range between 0 and 52. Finally, acne severity is graded as mild (GAGS: 1–18), moderate (GAGS: 19–30), severe (GAGS: 31–38), or very severe (GAGS >38).^46^

#### 2.3.2 Semistructured questionnaire and operational definitions of variables

A questionnaire gathering demographic, personal and clinical data was developed by the authors and cross-checked by a panel of experts in dermatology, pharmacology, and research methodology to ensure content validity. The questionnaire was subsequently piloted with 20 participants, administered by the authors, and revised for accuracy and appropriateness to ensure face validity on the basis of the qualitative feedback of the participants. The questionnaire is divided into three sections:

1. The first section included questions about demographic and personal characteristics, including age (3-level ordinal variables: 18–19, 20–21, ≥ 22 years); gender; study major; year of study (1st to 6th); self-reported weight (in kilograms) and height (in meters); smoking status (current smoker as smoking cigarettes, shisha and/or vape on some days during the last year; previous smoker as participant who quit smoking more than one year before the study; or nonsmoker).
  BMI was calculated on the basis of self-reported values of weight and height. The participants were assigned to BMI categories on the basis of the World Health Organization (WHO) BMI classification: < 18.5 kg/m^2^ was classified as underweight, 18.5–24.9 kg/m^2^ was classified as normal weight, 25.0–29.9 kg/m^2^ was classified as overweight, and > 30.0 kg/m^2^ was classified as obese.^47^^,^^48^
2. The second section included questions on family history of acne (parental, sibling, negative family history); receipt of a professional diagnosis of acne; bodily location of acne (forehead, right cheek, left cheek; nose; chin; trunk); and duration of acne (<1 year, 1–5 years, 5–10 years, >10 years).
3. The third section assessed the use, forms, reasons for use, sources of information, responses to, and side effects of CAM. This section includes a list of local and global CAM forms suggested by the authors in consultation with pharmacology professors. The list was divided into five types^49^:

- Biological forms of CAM include herbs; plant-based and animal-based products that are used locally; vitamins (vitamins B and C; multivitamin supplements); oily products; chelation therapy; and diet (low-glycemic, keto diet, etc.).
- Practices that are based on mind–body interactions include relaxation, yoga, meditation, and hypnotherapy.
- Body-based practices included cupping therapy, reflexology, massage, and chiropractic therapy.
- The entire medical system included Chinese medicine and prophetic medicine.
- Energy medicine includes acupuncture, magnets, and therapeutic touch.

### 2.4 Data analysis

Data analysis was performed via the Statistical Package for Social Sciences software version 26 (SPSS). Age was reported as the mean (±*SD*) and frequency after its categorization into an ordinal variable. Other categorical and ordinal variables are reported as frequencies and percentages. Overall GAGS and between-group differences in GAGS are reported as medians and mean ranks. The total percentage of certain variables exceeded 100 % due to the possibility of multiple responses. These variables are the bodily location of the acne, the medications prescribed to treat acne, the form of CAM, the reason for using CAM, the source of information, and the side effects. The Shapiro–Wilk test was used to examine the normality of the GAGS.^50^ A *p* value of less than 0.05 was considered to indicate statistical significance. The Mann–Whitney U and Kruskal–Wallis tests were employed to assess statistically significant differences in GAGS scores between two and three or more groups, respectively.^51^^,^^52^ A multivariate analysis was conducted using a multiple linear regression model that included the factors demonstrating significant associations at the bivariate level. To handle missing data, listwise deletion was used for cases with more than one missing variable.^53^

### 2.5 Ethical considerations

Approval was obtained from the xxx. This study adhered to the guidelines of the xxx. The clinical examination was carried out with privacy, dignity, and confidentiality. The objectives and conduct of the study, including the clinical examinations involved, were clearly communicated to potential participants by the examiner and interviewer before providing verbal informed consent and agreeing to participate in the study.

## 3 Results

### 3.1 Background characteristics of the study sample

A total of 202 out of 402 participants were diagnosed with acne, resulting in a prevalence rate of 50.2 %. The final sample consisted of 190 participants, as 11 participants were excluded because of missing data (5.4 %). The sample comprised 144 females (75.8 %) and 46 males (24.2 %), with a mean age of 20.4 years (*SD* ± 1.6). Most participants were studying medicine (63.7 %), followed by nursing (6.8 %), dentistry (5.8 %) and medical imaging (4.7 %). The majority were either in their first (28.9 %) or second (26.8 %) year of study. Approximately two-thirds had a BMI within the healthy range (65.7 %). Most participants were nonsmokers (86.3 %), whereas only a minority were current smokers (12.1 %) (see Table 1 for more details on the background characteristics of the participants).

**Table 1:** Background characteristics of the respondents.

Demographic factors	N (%)
**Age in years**	
18–19	66 (34.7)
20–21	67 (35.3)
≥ 22	57 (30.0)

**Gender**	
Male	46 (24.2)
Female	144 (75.8)

**BMI**	
Underweight	21 (11.6)
Healthy range	119 (65.7)
Overweight	29 (16.0)
Obese	12 (6.6)

**Study Major**	
Medicine	121 (63.7)
Nursing	13 (6.8)
Dentistry	11 (5.8)
Medical imaging	9 (4.7)
Pharmacy	8 (4.2)
Physiotherapy	7 (3.7)
Anesthesia and Resuscitation	6 (3.2)
Optometry	3 (1.6)
Midwifery	3 (1.6)
Medical Laboratory Sciences	3 (1.6)
Speech Pathology	2 (1.1)
Pharmacy Doctor	2 (1.1)
Cardiac perfusion technology	1 (0.5)
Cosmetics and Skin Care	1 (0.5)

**Year of study**	
First year	55 (28.9)
Second year	51 (26.8)
Third year	29 (15.3)
Fourth year	27 (14.2)
Fifth year	21 (11.1)
Sixth year	7 (3.7)

**CAM use**	
Yes	119 (62.6)
No	71 (37.4)

**Smoking history**	
Current smoker	23 (12.1)
Previous smoker	3 (1.6)
Nonsmoker	164 (86.3)

**Milk consumption**	
Most days	77 (41.8)
1–2 per week	67 (36.4)
None	40 (21.7)

Abbreviations: BMI: body mass index, CAM: complementary and alternative medicine.

### 3.2 Acne-related clinical characteristics

By employing the Shapiro–Wilk test, the GAGS score was found to be nonnormally distributed (*p* < 0.001). The median severity score was 10, with 77.4 % having mild acne, 17.9 % having moderate acne, 3.7 % having severe acne, and only 1.1 % having very severe acne. Among the 156 (82.1 %) participants who reported having a positive family history, 34 (17.9 %) had a parental family history, and 136 (71.6 %) had a sibling family history. Nearly one-third had acne for less than one year (35.3 %), and approximately half of the participants had acne for one to five years (50.5 %). Most participants had acne on the forehead (62.6 %), followed by an equal number of participants with acne on the left cheek and/or right cheek (56.8 %, each), chin (38.4 %), trunk (27.9 %), and nose (14.2 %) (see Table 2 for more details on the characteristics of the participants).

**Table 2:** Association between the demographic, personal, and clinical characteristics of the respondents and the severity of acne.

Factors	Frequency (%)	Median	Mean Rank	*P* value (bivariate analysis)	Multivariate analysis	
					*P* value	Regression coefficient
**Age in years**						
18–19	66 (34.7)	10.00	97.89	0.84	–	–
20–21	67 (35.3)	10.00	96.01			
≥ 22	57 (30.0)	10.00	92.13			

**Gender**						
Male	46 (24.2)	8.00	86.45	0.19	–	–
Female	144 (75.8)	11.00	98.39			

**BMI**						
Underweight	21 (11.6)	8.00	86.24	0.44	–	–
Healthy range	119 (65.7)	12.00	95.34			
Overweight	29 (16.0)	8.00	82.84			
Obese	12 (6.6)	7.00	75.96			

**Family history**						
Positive family history	156 (82.1)	12.00	100.85	0.004⁎	0.284	1.671
Negative family history	34 (17.9)	7.00	70.94			

**Study major**						
Medicine	121 (63.7)	10.00	94.01	0.69	–	–
Nursing	13 (6.8)	10.00	96.73			
Dentistry	11 (5.8)	9.00	91.45			
Pharmacy	8 (4.2)	10.50	84.00			
Medical imaging	9 (4.7)	8.00	86.44			
Physiotherapy	7 (3.7)	10.00	105.21			
Anesthesia and Resuscitation	6 (3.2)	9.00	95.00			
Speech Pathology	2 (1.1)	22.50	154.75			
Pharmacy Doctor	2 (1.1)	4.50	37.50			
Medical Laboratory Sciences	3 (1.6)	21.00	121.00			
Midwifery	3 (1.6)	9.00	110.50			
Optometry	3 (1.6)	15.00	188.67			
Cardiac perfusion technology	1 (0.5)	16.00	129.00			
Cosmetics and Skin Care	1 (0.5)	33.00	185.50			

**Year of study**						
First year	55 (28.9)	15.00	108.31	0.06	–	–
Second year	51 (26.8)	7.00	82.22			
Third year	29 (15.3)	8.00	88.47			
Fourth year	27 (14.2)	15.00	112.43			
Fifth year	21 (11.1)	10.00	88.64			
Sixth year or more	7 (3.7)	8.00	76.07			

**Professional diagnosis**						
Yes	79 (41.6)	15.00	114.73	<0.001⁎	0.171	2.166
No	111 (58.4)	8.00	81.82			

**OTC use**						
Yes	59 (31.3)	18.00	123.17	<0.001⁎	0.009⁎	4.381
No	131 (68.9)	8.00	83.04			

**CAM use**						
Yes	119 (62.6)	10.00	97.38	0.54		
No	71 (37.4)	10.00	92.35			

**Duration of Acne**						
Less than a year	67 (35.3)	6.00	71.01	<0.001⁎	<0.001⁎	4.726
1–5 years	96 (50.5)	12.00	99.67			
5–10 years	24 (12.6)	21.00	139.10			
More than 10 years	3 (1.6)	27.00	160.00			

**Smoking**						
Yes	26 (13.7)	12.00	92.58	0.77	–	–
No	164 (86.3)	9.50	95.96			

**Milk Consumption**						
Most days	77 (41.8)	12.00	95.34	0.65		
1–2 times per week	67 (36.4)	9.00	93.20			
None	40 (21.7)	8.00	85.85			

Abbreviations: BMI: body mass index, OTC: over the counter.

⁂ Frequency (percentages) of missing values: BMI 9 (4.7 %) - milk consumption 6 (3.1 %).

⁎ : *p* value is below the threshold for significance (0.05).

Fewer than half of the patients said that their acne was diagnosed by a dermatologist (41.6 %). The most frequently prescribed medication was isotretinoin (19.3 %), followed by oral antibiotics (2.5 %) and topical pharmaceutical preparations (5.7 %). Approximately one-third of the participants used OTC products (31.1 %), with facial cleansers as the most common product (48.2 %), followed by creams and moisturizers (30.7 %) and cleansing soap (21.1 %).

### 3.3 Findings on CAM use

Among CAM users (62.6 %), the most prevalent forms were blossom water (35.3 %), *aloe vera* (33.6 %), yogurt (26.9 %), honey (25.2 %), massage (23.5 %), rice water (19.3 %), and lime (16.8 %) (Table 3). Nearly half of the CAM users cited a perceived lack of severity as the main reason for using CAM (52.1 %). Other reasons were seeking online solutions (34.5 %), following people's recommendations (29.4 %), convenience of obtaining CAM (16.0 %), and having no time for professional consultation (15.1 %). Social media (47.9 %) was the most common source of information, followed by the internet (46.2 %), family (38.7 %), friends (27.7 %), and healthcare professionals (23.5 %) (see Table 4 for more details). While 39.5 % of CAM users reported perceived benefits from CAM use, 44.5 % reported no significant impact. Dehydration (34.5 %), erythema (31.1 %), and desquamation (24.4 %) were the most reported side effects of CAM use (see Table 5 for details on the response to CAM use).

**Table 3:** Methods of complementary and alternative medicine.

CAM Name	Frequencies (%)
**Biological Forms**	
Blossom water	42 (35.3)
*Aloe vera*	40 (33.6)
Yogurt	32(26.9)
Honey	30 (25.2)
Rice water	23 (19.3)
Lime	20 (16.8)
Egg	11 (9.2)
Cinnamon	9 (7.6)
Turmeric	9 (7.6)
Yeast	8 (6.7)
Parsley	7 (5.9)
Vinegar	6 (5.0)
Tea	6 (5.0)
Rosemary	6 (5.0)
Mint	5 (4.2)
Oats	2 (1.7)
Orange	2 (1.7)
Georgina	1 (0.8)
Multivitamins	15 (12.6)
Vitamin C	17 (14.3)
Vitamin B12	3 (2.5)
Vitamin E	3 (2.5)
Intermittent fasting diet	2 (1.7)
Low-carb diet	8 (6.7)
Keto diet	1 (0.8)
Oily products	13 (10.9)

**Mind-body medicine**	
Relaxation	19 (16.0)
Meditation	15 (12.6)
Yoga	10 (8.4)

**Whole medical systems**	
Prophetic medicine	7 (5.9)
Chinese medicine	2 (1.7)

**Body-based practices**	
Massage	28 (23.5)
Wet cupping	9 (7.6)

**Table 4:** Reasons for and sources of information on complementary and alternative medicine.

Pattern of CAM use	N (%)
**Reasons for using CAM:**	
Lack of severity	62 (52.1)
Self-treatment using the internet	41 (34.5)
Others' recommendations	35 (29.4)
Convenience of using CAM	19 (16.0)
No time for seeking medical advice	18 (15.1)
Recommendation made by a pharmacist	15 (12.6)
Safety of CAM	13 (10.9)
Previous use of CAM	12 (10.1)
Professional treatment is costly	10 (8.4)
Embarrassment resulting from seeking professional advice	5 (4.2)

**Source of information on CAM use:**	
Internet	55 (46.2)
Social Media	57 (47.9)
Family	46 (38.7)
Friends	33 (27.7)
Healthcare professional	28 (23.5)
Commercials	12 (10.1)
Books/magazines	6 (5.0)
Other persons	3 (2.5)
TV	2 (1.7)
Articles	2 (1.7)

**Table 5:** Responses and side effects of using complementary and alternative medicine.

Pattern of CAM use	N (%)
**The responses noticed after using CAM:**	
Better effect	47 (39.5)
Worse effect	19 (16.0)
No effect	53 (44.5)

**Side effects from using CAM:**	
Dehydration	41 (34.5)
Erythema	37 (31.1)
Desquamation	29 (24.4)
Itching	20 (16.8)
Eye itching	8 (6.7)
Eye redness	7 (5.9)
Swelling	6 (5.0)
Skin discolouration	5 (4.2)
Hair changes	4 (3.4)
Ulcers	4 (3.4)
Nail changes	3 (2.5)
Fever	2 (1.7)
Scaling	1 (0.8)

### 3.4 Associations between acne severity and other variables

At the bivariate level, acne severity was associated with OTC product use (*p* *<* 0.001), professional diagnosis *(p* < 0.001*),* duration of acne (*p* < 0.001), and family history of acne (*p* = 0.004). The other demographic, personal and clinical variables were not associated with acne severity. Furthermore, OTC product use was associated with receiving a professional diagnosis (*p* < 0.001), whereas no association was found between CAM use and receiving a professional diagnosis (*p* = 0.630). At the multivariate level, only OTC use (*p* = 0.009) and duration of acne (*p* < 0.001) remained significant, whereas professional diagnosis *(p* = 0.171*)* and family history of acne (*p* = 0.284) did not demonstrate statistical significance (Table 2).

## 4 Discussion

This cross-sectional study, which was conducted among university students, revealed that the prevalence of acne was 50.2 %. Among the participants diagnosed with acne, approximately one-third used OTC products, whereas nearly two-thirds used CAM, with social media and the internet as the main sources of information. Acne severity was associated with both OTC use and the duration of acne. Additionally, receiving a professional diagnosis was associated with OTC use but not with CAM use. The majority of the study sample consisted of females, probably due to their interest in skin care, distinct health-seeking behavior, and greater impact of acne. Studies have shown that females are more inclined to seek dermatological consultation for acne^54^ and to use skin care products.^55^ Additionally, this study reported a prevalence rate higher than that used for sample size calculation, which should be considered in future studies. The prevalence rate of acne varies across regional studies targeting similar age populations and using clinical diagnosis for case ascertainment, with rates of 34.7 % in Syria, 40.1 % in Turkey, and 56.2 % in Saudi Arabia.^56^, ^57^, 58.

Nearly one-third of the participants in this study reported using OTC products, which correlated with both acne severity and receiving a professional diagnosis. The use of OTC products for acne varies across regions. For example, one study in Saudi Arabia reported use rates of 44.3 % and 13.9 % for cleansers and leave-on products, respectively.^59^ Another study in Turkey reported lower rates of 10.6 % for cleansers and 2.5 % for moisturizers.^2^ Although OTC products are available without a prescription, most users in this study received a professional diagnosis of acne, which suggests that these products may have been recommended by dermatologists rather than used as a form of self-care.

This study revealed that neither clinical severity nor professional diagnosis was associated with CAM use. Notably, CAM use was reported by most participants, with a lack of severity cited as the most common reason for CAM use. The CAM use rate revealed by this study (62.6 %) falls within the high range of regional rates (56.9–77 %).^2^^,^60., 61., 62, 63 Given the lack of temporal assessment in this cross-sectional study, it is unclear whether CAM was used before or after seeking professional care. This suggests a need to address CAM use during clinical consultations, as patient–physician interactions represent crucial decision-making points where mutual discussions can encourage safe and effective use. Future studies should explore patterns of CAM and OTC product use along the care pathway, examining their use before and after medical consultations.

However, access to specialized dermatological services is often limited by multiple factors, including social, financial, and systemic barriers.^64^^,^^65^ In the context of acne, sociocultural norms may influence perceptions of the condition as mild or transient, encouraging the use of CAM and, in some cases, delaying professional treatment.^66^^,^^67^ Indeed, the participants in this study cited a lack of severity as the most common reason for using CAM. Moreover, limited access to dermatologists, whether due to shortages or inequitable distributions, is a global challenge, even in high-income countries.^68^

The findings of this study provide insights that may inform interventions in both clinical practice and public health by addressing the most commonly used OTC products and CAM approaches, their utilization patterns, and sources of information. While health-seeking behaviors and barriers to access may delay formal treatment, CAM and OTC product use itself is not inherently problematic and should be integrated into the acne care pathway. In clinical practice, healthcare professionals should discuss self-care practices with patients, adopting a shared decision-making approach that respects patient preferences while ensuring safe and effective care. Integrating safe elements of traditional medicine into acne management may improve patient adherence and treatment outcomes. This culturally sensitive approach is especially relevant to Palestine, where previous studies have shown a high preference rate for CAM use across different patient populations and age groups.^69^, ^70^, ^71^ In community settings, educational interventions can help improve self-care practices by promoting informed decision-making and safe, effective use of self-care methods. Moreover, understanding how people access health information can improve the effectiveness of educational interventions. Given that the participants in this study turn to the internet and social media for medical information, using these channels in designing communication strategies can enhance the reach, engagement, and impact of these interventions. Notably, previous studies have also highlighted the popular use of online sources in CAM-related acne management worldwide, emphasizing the need for digital health strategies.^60^^,^^63^

This study found no associations between acne severity and demographic, genetic, or personal factors. Heng and Chew's systematic review concluded that while most studies have reported associations between male sex, age, and BMI, on the one hand, and acne severity, on the other hand, considerable research has yielded mixed evidence.^72^ For example, evidence for an association between male sex and moderate to severe acne remains unsubstantiated.^73^, ^74^, ^75^, 76., 77 Additionally, most studies reporting an association between age and acne severity are limited to adolescent populations,^76^^,^^78^^,^^79^ whereas the present study included an older population of university students.

## 5 Strengths and limitations

This study contributes to the relatively limited dermatology research in Palestine. By identifying patterns of self-care in acne patients, the findings of this study provide hypotheses informing further research and health interventions. Nonetheless, the major limitation of this study is the small sample size due to conflict-related safety and mobility concerns, which might have undermined its statistical power. The second limitation is that the study did not exclude participants who were underweight or overweight. However, no association was found between BMI and acne severity at the bivariate level in this study, which is consistent with the inconclusive evidence for the impact of BMI on acne severity.^80^^,^^81^ Moreover, as this research was conducted at the university campus where logistical constraints limit the use of weight and height scales in the examination room, self-reported data on weight and height were used to calculate BMI. Furthermore, the generalizability of the findings is limited by the characteristics of the sample. University health science students, whose health and disease behaviors may differ from those of the general population, were the focus of the study. University students, especially those studying health-related majors, might be more inclined to resort to evidence-based treatments than the general population is. Future studies should assess knowledge about CAM and OTC products via a knowledge questionnaire with a scoring system. This would allow for a better understanding of how knowledge levels may influence treatment choices and patterns of product use, especially among health students.

## 6 Conclusions

This study aimed to explore self-care methods, including OTC products and CAM, among health students diagnosed with acne and to assess the associations between the use of these methods and acne severity. Nearly one-third of the participants used OTC products, whereas almost two-thirds used CAM, predominantly relying on the internet and social media for information. OTC product use was associated with both acne severity and receiving a professional diagnosis, whereas CAM use showed no such associations. Moreover, clinical severity was associated with acne duration. Future studies are recommended to explore discussions of acne self-care in clinical consultations and the timing of the use of self-care methods along the acne care pathway. In clinical practice, healthcare professionals should address self-care methods via a shared decision-making approach that considers patient preferences and safety. At the community level, the forms and patterns of self-care behaviors and the preferred communication channels can guide educational interventions to empower acne patients with informed decision-making, ensuring that self-care methods are safe and effective.

## CRediT authorship contribution statement

**Sari Taha:** Writing – review & editing, Writing – original draft, Supervision, Methodology, Formal analysis, Data curation, Conceptualization. **Manal Taha:** Writing – review & editing, Writing – original draft, Validation, Formal analysis, Data curation. **Sa’ed H. Zyoud:** Writing – review & editing, Writing – original draft, Supervision, Project administration, Methodology, Formal analysis, Conceptualization.

## Declaration of competing interest

The authors declare that they have no known competing financial interests or personal relationships that could have appeared to influence the work reported in this paper.
